# Blood B Lymphocyte Stimulator (BLyS)/BAFF levels may reflect natural immunity to HIV in highly exposed uninfected Beninese Commercial Sex Workers

**DOI:** 10.1038/srep32318

**Published:** 2016-08-26

**Authors:** Catherine Sabourin-Poirier, Lyvia Fourcade, Josiane Chagnon-Choquet, Annie-Claude Labbé, Michel Alary, Fernand Guédou, Johanne Poudrier, Michel Roger

**Affiliations:** 1 Laboratoire d’immunogénétique, Centre de Recherche du Centre Hospitalier de l’Université de Montréal (CRCHUM), Montréal, Canada; 2 Département de Microbiologie, Infectiologie et Immunologie de l’Université de Montréal, Montréal, Canada; 3 Centre de recherche du CHU de Québec, Québec, Canada; 4 Département de médecine sociale et préventive, Université Laval, Québec, Canada; 5 Dispensaire des IST, Cotonou, Benin

## Abstract

We have previously shown that excess B lymphocyte Stimulator (BLyS)/BAFF in plasma and on surface of blood dendritic cells (DC) of HIV-infected progressors coincides with B-cell dysregulations and increased frequencies of “precursor” innate marginal zone (MZ)-like B-cells. In contrast, both blood BLyS levels and frequencies of this population remained unaltered in HIV elite-controllers. Based on these observations, we hypothesized that control of BLyS and innate B-cell status could be associated with natural immunity against HIV infection. Therefore, we assessed blood BLyS levels and B-cell status in HIV highly-exposed commercial sex workers (CSWs) from Benin. We found blood BLyS levels of HIV-uninfected CSWs were lower than those observed in both HIV-infected CSW and HIV-uninfected non-CSW groups. Furthermore, levels of BLyS expression on blood T-cells and monocytes were lower in HIV-uninfected CSWs when compared to HIV-infected CSWs, but higher than those observed for HIV-uninfected non-CSWs. Concomitantly, HIV-infected CSWs presented a dysregulated blood B-cell compartment, characterized by increased total IgG1, increased frequencies of populations presenting immature and/or innate profiles and a higher ratio of IgG^+^/IgA^+^ plasmablasts. In contrast, relatively low levels of BLyS in the blood of HIV-uninfected CSWs coincided with a rather preserved B-cell compartment.

Worldwide, most HIV infections are acquired through heterosexual intercourse, and in sub-Saharan Africa, 60% of new HIV infections affect women^1^. Vaccines and microbicides hold promise for preventing the acquisition of HIV, and the success of designing such agents will benefit from the study of HIV highly-exposed seronegative (HESN) individuals, who provide a model of natural immunity to HIV. High levels of anti-inflammatory and neutralizing proteins, such as anti-proteases and HIV-specific immunoglobulins (Igs) are found in the genital mucosa of HESN^2^,^3^. In a cohort of HESN women from Ivory Coast, HIV-specific mucosal IgA were shown to block viral transcytosis through tight epithelial barriers^3^,^4^,^5^. In a Kenyan female commercial sex worker (CSW) cohort, HIV-specific CD4^+^ and CD8^+^ T-cell responses as well as cross-clade neutralizing IgA have been found in both the blood and genital tract of HESN CSWs^2^,^3^,^6^,^7^,^8^,^9^,^10^. In these individuals a low activation T-cell profile corresponds with a greater ability to proliferate in response to HIV p24 peptides when compared to HIV-infected CSWs^11^. Furthermore, elevated frequencies of T-regulatory lymphocytes have been found in the blood of HESN CSWs^12^. In addition, we have previously shown that Beninese female HESN CSWs had significantly lower genital levels of pro-inflammatory cytokines such as TNF-α and IFN-γ than HIV-infected CSWs^13^. Altogether, these findings suggest that the capacity to maintain a low-key activation/inflammatory profile is associated with protection against HIV infection.

Until now, few studies have assessed B-cell expression profiles in the context of natural immunity against HIV. The detailed characterization of the Ig repertoire of cervical and systemic B-cells from a Kenyan HESN individual revealed that site-specific responses occur with unique regulation of tolerance and recruitment into local memory or blast B-cell compartments, and the infusion of systemic post-germinal center (GC) B-cells to the cervix seems to be a common event^14^. Understanding the nature and how these B-cell populations are solicited appears important to the design of preventive approaches.

Although the specific factors responsible for the natural immunity against HIV have yet to be fully unraveled, we believe that observations from HIV elite-controllers (EC) can shed some light. As such, our previous studies suggest that control of HIV disease progression may be linked to B lymphocyte Stimulator (BLyS)/BAFF expression status, and to its capacity of orchestrating B-cell population dynamics and responses^15^. Indeed, we have shown that BLyS over-expression in the blood of HIV-1-infected progressors coincided with major B-cell dysregulations and hyperglobulinemia, with increased frequencies of an activated population presenting characteristics of both transitional immature and innate marginal zone (MZ)-like B-cells, designated as “precursor MZ-like”^16^,^17^. In contrast, in EC, BLyS levels and precursor MZ-like B-cell frequencies remained similar to those observed in HIV-negative donors. Rather, percentages of MZ-like B-cells presenting a more “mature” profile were decreased when compared to both HIV progressors and HIV-negative individuals^16^,^17^. These findings suggest that the presence of these cells in a preserved BLyS non-inflammatory environment, such as encountered in EC, could be beneficial to the battle and even control of HIV. In an effort to further unravel elements associated with natural immunity to HIV, we have assessed blood BLyS levels and B-cell status in female CSWs from Benin.

## Results

### Socio-demographic characteristics of the study population

The socio-demographic characteristics of female CSWs and non-CSWs are shown in Table 1. The three study groups were similar with respect to age and vaginal douching practice. Duration of sex work, average number of clients and condom use were similar between the HIV-infected and HIV-uninfected CSW groups.

### Levels of expression of BLyS in serum and on blood T-cells, monocytes, myeloid dendritic cells (mDC) of HIV-uninfected CSWs, HIV-infected CSWs, and HIV-uninfected non-CSWs

**Figure 1:**
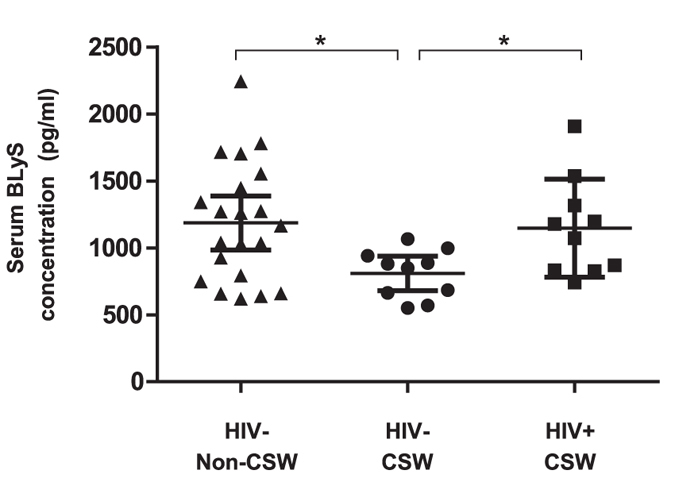
Concentration of BLyS in the serum of HIV-uninfected non-commercial sex workers (CSWs), HIV-uninfected CSWs and HIV-infected CSWs. Serum BLyS concentrations (pg/ml) as mean ± SEM were compared with unpaired T test for pair-wise comparisons between HIV-uninfected CSWs and the two other groups. Significance levels are shown as *(p < 0.05). The mean BLyS serum level of the 10 non-CSWs used for the phenotype assays was 1152 pg/ml, which is similar to the mean of the whole non-CSW group (1186 pg/ml) shown here.

**Figure 2:**
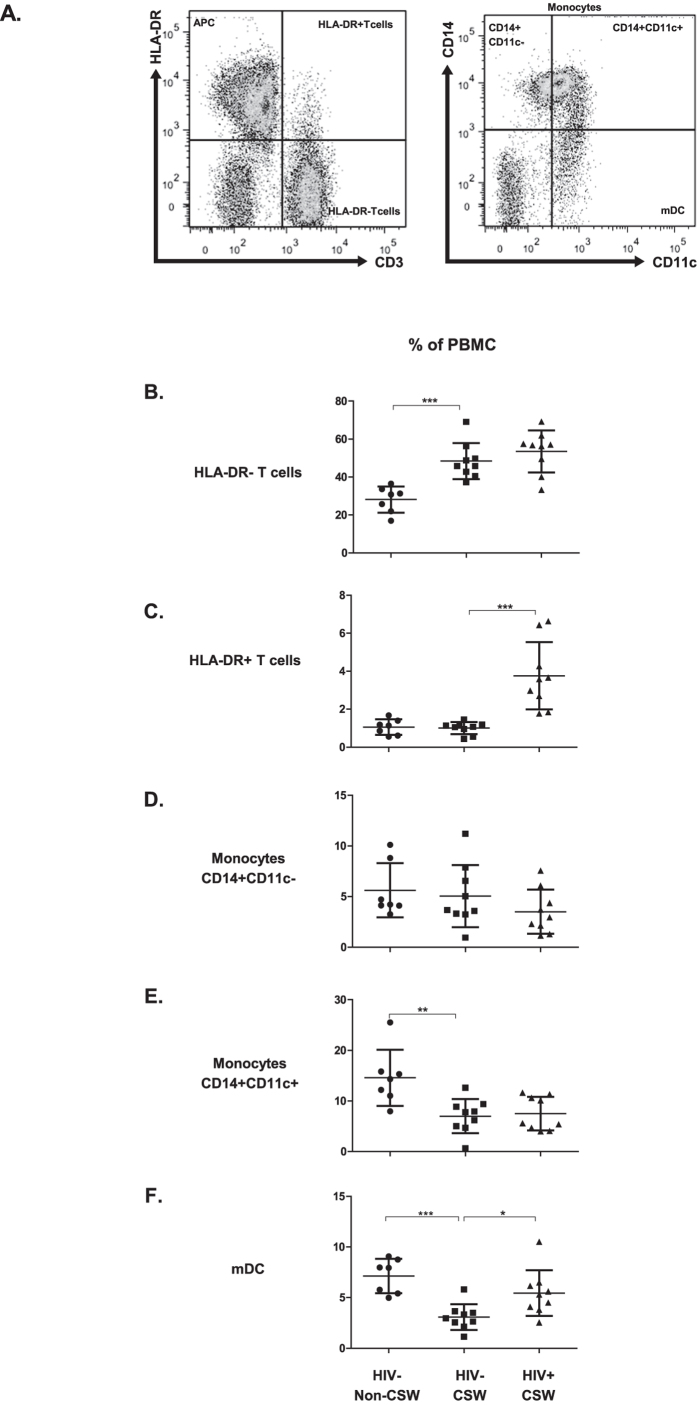
Frequencies of T-cells, monocytes, and myeloid DC (mDC) in the blood of HIV-uninfected non-commercial sex workers (CSWs), HIV-uninfected CSWs and HIV-infected CSWs. Analysis by flow-cytometry was performed as follows: (**A**) Cells were gated on live PBMCs and then on HLA-DR^-^CD3^+^ (HLA-DR^-^ T-cells), HLA-DR^+^ CD3^+^ (HLA-DR^+^ T-cells) and HLA-DR^+^CD3^−^ (APCs). APCs were further characterized as CD14^+^CD11c^-^ and CD14^+^CD11c^+^ monocytes, and total CD14^-^CD11c^+^ cells, which comprise a majority of myeloid DC (mDC). Quadrants were set based on the expression values obtained with fluorescence minus one (FMO) and isotype controls. Frequencies (%) of (**B**) HLA-DR^−^ T-cells, (**C**) HLA-DR^+^ T-cells cells, (**D**) CD14^+^CD11c^−^ monocytes, (**E**)CD14^+^CD11c^+^ monocytes, and (**F**) mDC are relative to live PBMCs. The mean events gated for these populations are in the range of: CD14+CD11c- monocytes (1490 ± 625), CD14+CD11c+ monocytes (9892 ± 962), total mDC (CD14-CD11c+) (1004 ± 300). Representative FMO staining controls can be viewed in Supplementary Fig. S3. Cell population percentages were compared with the Mann-Whitney U tests for pair-wise comparisons between HIV-uninfected CSWs and the two other groups. Significance levels are shown as *(p < 0.05), **(p < 0.01), ***(p < 0.001).

BLyS levels measured in serum of HIV-uninfected CSWs were significantly lower than those observed in HIV-infected CSWs and HIV-uninfected non-CSWs (Fig. 1). Because determining and comparing frequencies of cells expressing BLyS might be influenced by the fluctuations in cell populations between the study groups^16^,^18^, we have assessed the relative percentages of T-cells, monocytes and mDC in the blood of the three study groups. The percentages of HLA-DR^-^ T-cells were increased in the blood of both HIV-uninfected and HIV-infected CSWs when compared to HIV-uninfected non-CSWs (Fig. 2B) whereas the frequencies of activated HLA-DR^+^ T-cells were lower in the HIV-uninfected groups (Fig. 2C) when compared to HIV-infected CSWs. For consistency and comparison with our previously published observations 16,18,19, we have analyzed the minor fraction of CD14^+^ monocytes which do not express detectable levels of CD11c, separately from the bulk of CD14^+^CD11c^+^ monocytes. In fact, when compared to CD14+CD11c- monocytes, CD14+CD11c+ cell percentage greatly fluctuated in HIV+ progressors^18^, and these cells expressed greater levels of surface BLyS^16^,^17^. For means of comparison with the current consensus on monocyte nomenclature, CD14+CD11c− and 90–95% of CD14 + CD11c+ monocytes are CD16-, and fall into the classical monocytes. A minor fraction of the CD14 + CD11c + monocytes are CD14lo and express CD16 (not shown), and fall into the non-classical nomenclature^20^. Although no significant differences were observed in frequencies of the minor CD14^+^CD11c^−^ monocytes between the three study groups (Fig. 2D), we found frequencies of CD14^+^CD11c^+^ monocytes were greatly diminished in the blood of both CSWs groups when compared to HIV-uninfected non-CSWs (Fig. 2E). Frequencies of total CD14-CD11c+ cells, which comprise a majority of myeloid DC (mDC)^20^,^21^, and will be referred to mDC throughout the manuscript, were lower in HIV-uninfected CSWs when compared to both HIV-infected CSWs and HIV-uninfected non-CSWs (Fig. 2F).

**Figure 3:**
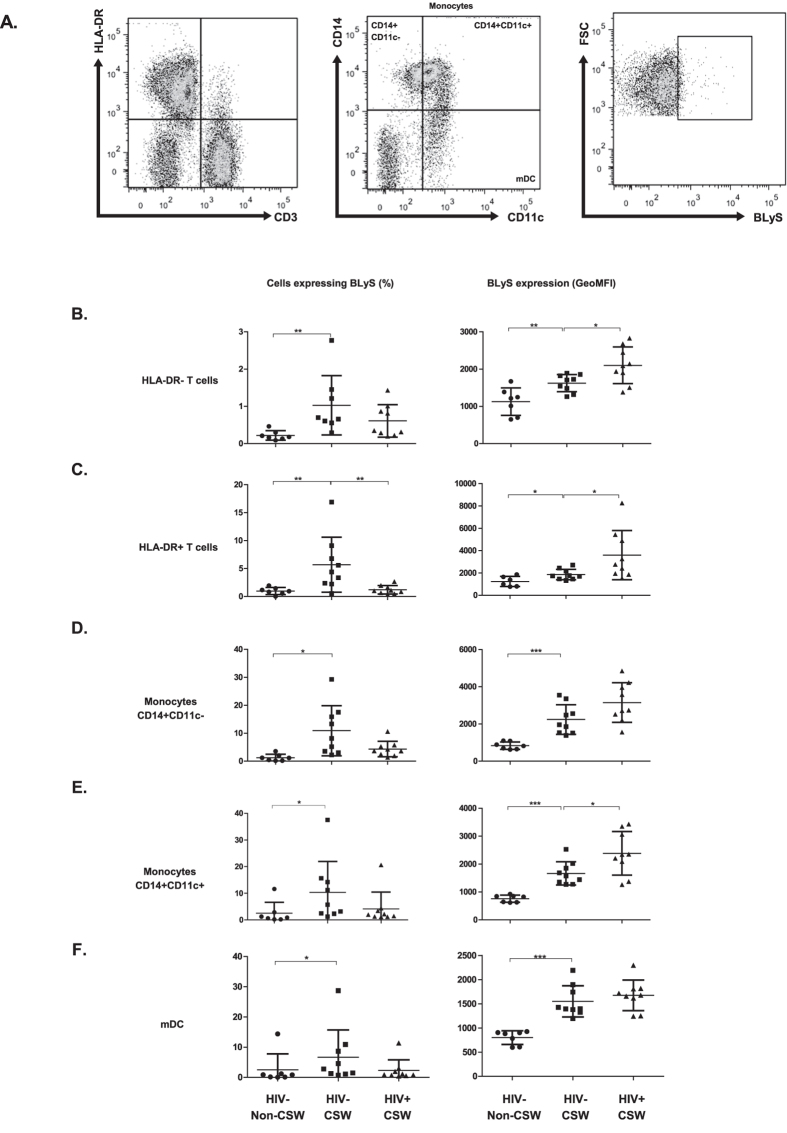
BLyS surface expression levels by T-cells, monocytes, and myeloid DC (mDC) in the blood of HIV-uninfected non-commercial sex workers (CSWs), HIV-uninfected CSWs and HIV-infected CSWs. Analysis by flow-cytometry was performed as follows: (**A**) Cells were gated on live PBMCs and then on HLA-DR^-^CD3^+^ (HLA-DR^-^ T-cells), HLA-DR^+^CD3^+^ (HLA-DR^+^ T-cells) and HLA-DR^+^CD3^−^ (APC). APCs were further characterized as CD14+CD11c- and CD14+CD11c+ monocytes, and mDC (CD14^-^CD11c^+^). Quadrants were set based on the expression values obtained with fluorescence minus one (FMO) and isotype controls. Percentages (%) of cells expressing BLyS (left panels) and Geometric mean fluorescence intensity (GeoMFI) of membrane BLyS expression levels (right panels) are relative to each following population: (**B**) HLA-DR^-^ T-cells, (**C**) HLA-DR^+^ T-cells cells, (**D**) CD14+CD11c-monocytes, (**E**)CD14+CD11c+ monocytes, and (**F**) CD14-CD11c+ cells (mostly mDC). Representative FMO staining controls can be viewed in Supplementary Fig. S3. Percentages of cells expressing BLyS and levels of BLyS expression (GeoMFI) were compared with the Mann-Whitney U tests for pair-wise comparisons between HIV-uninfected CSWs and the two other groups. Significance levels are shown as *(p < 0.05), **(p < 0.01), ***(p < 0.001).

Taking into account these observations, we found significantly higher relative frequencies of T-cells, monocytes and mDC expressing BLyS in the blood of HIV-uninfected CSWs when compared to those observed in HIV-uninfected non-CSWs, and a similar trend when comparing to those of HIV-infected non-CSWs (Fig. 3B–F, left panels). BLyS surface expression levels for these cell populations in HIV-uninfected CSWs were higher than those observed in HIV-uninfected non-CSWs but lower than those observed in HIV-infected CSWs (Fig. 3B–F, right panels). Overall, HIV-uninfected (HESN) CSWs had the lowest levels of BLyS in their serum, but presented greater frequencies of BLyS expressing cells in their blood when compared to both HIV-uninfected non-CSWs and HIV-infected CSWs. However, HIV-infected CSWs presented the highest levels of BLyS cell surface expression.

### Analysis of B-cell populations in the blood of HIV-uninfected CSWs, HIV-infected CSWs, and HIV-uninfected non-CSWs

**Figure 4:**
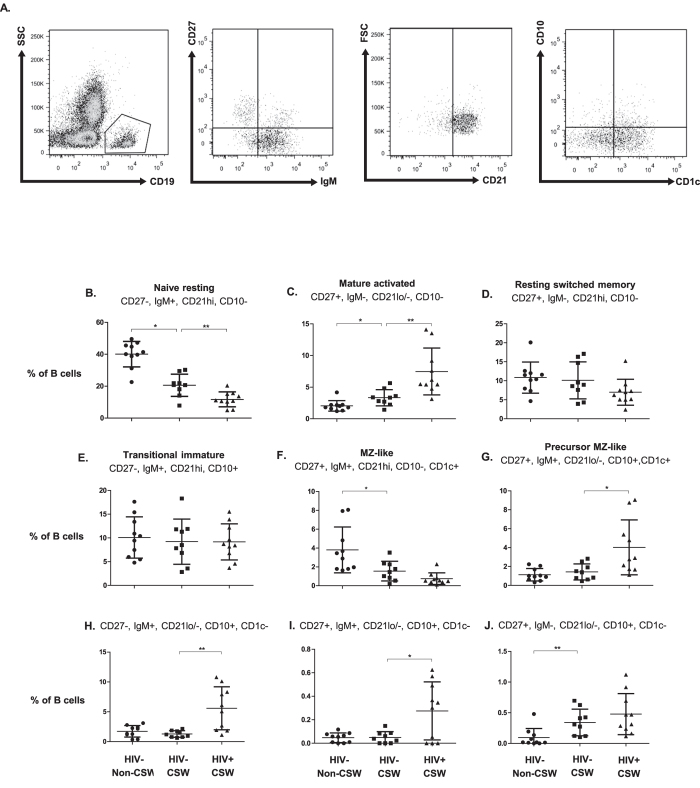
B-cell populations in the blood of HIV-uninfected non-commercial sex workers (CSWs), HIV-uninfected CSWs and HIV-infected CSWs. Analysis by flow-cytometry was performed as follows: (**A**) Cells were gated on live PBMCs and then on total B-cells (CD19^+^). Total CD19^+^ B-cells were selected based on expression of CD27 and/or IgM, and then on their expression levels of CD21. CD1c and CD10 expression were used for further characterization of blood marginal zone (MZ) and transitional immature (TI) B-cell populations. Quadrants were set based on the expression values obtained with fluorescence minus one (FMO) and isotype controls. Naïve resting B-cells were defined as CD19+CD27-IgM+CD21hiCD1c-CD10-, Mature activated B-cells were defined as CD19+CD27+IgM-CD21loCD1c-CD10-, resting switched memory B-cells were CD19+CD27+IgM-CD21hiCD1c-CD10-, transitional immature (TI) B-cells were CD19+CD27-IgM+CD21hiCD1c-CD10+, mature MZ-like B-cells were CD19+CD27+IgM+CD21hiCD1c+CD10-, precursor marginal-zone (MZ)-like B-cells were CD19+CD27+IgM+CD21loCD1c+CD10+. The Frequencies (%) of (**B**) naïve resting, (**C**) mature activated, (**D**) resting switched memory, (**E**) TI, (**F**) mature MZ-like, (**G**) precursor MZ-like, (**H**) (CD27^-^IgM^+^CD21^lo^CD10^+^CD1c^−^), (**I**) (CD27^+^IgM^+^CD21^lo^CD10^+^CD1c^-^), and (**J**) (CD27^+^IgM^-^CD21^lo^CD10^+^CD1c^−^). B-cell populations and/or differentiation stages are relative to total B-cells. The mean events gated for these populations are in the range of: total B-cells (9320 ± 1750), naïve (3728 ± 1045), mature activated (360 ± 67), resting switched memory (632 ± 301), TI (944 ± 174), mature MZ-like (327 ± 233), precursor MZ-like (145 ± 36). Representative FMO staining controls can be viewed in Supplementary Fig. S4. B-cell population percentages were compared with the Mann-Whitney U tests for pair-wise comparisons between HIV-uninfected CSWs and the two other groups. Significance levels are shown as *(p < 0.05), **(p < 0.01).

Relative percentages of total B-cells were lower in the blood of both HIV-uninfected and HIV-infected CSWs when compared to that of control non-CSWs (not shown). Naïve resting B-cell frequencies were higher in HIV-uninfected CSWs when compared to those in HIV-infected CSWs, but lower than those found in HIV-uninfected non-CSWs (Fig. 4B). Conversely, the frequency of mature activated B-cells was lower in HIV-uninfected CSWs than in HIV-infected CSWs, but higher than in HIV-uninfected non-CSWs (Fig. 4C). No significant difference in the frequencies of resting switched memory and transitional immature B cells were observed (Fig. 4D,E). As for mature MZ-like B-cells, frequencies were decreased in the blood of HIV-uninfected CSWs when compared to HIV-uninfected non-CSWs (Fig. 4F). As stated earlier, we had previously described increased frequencies of precursor MZ-like B-cells presenting an activated phenotype in the blood of HIV-infected progressors^16^,^17^. As such, we found a significant increased frequency of B-cells presenting this phenotype in the blood of HIV-infected CSWs when compared to the HIV-uninfected CSWs (Fig. 4G), we also found higher frequencies of B-cells presenting similar IgM^+^CD10^+^CD21^−/lo^ and GC-like CD27^+^IgM^−^CD10^+^CD21^−/lo^ characteristics in the blood of HIV-infected CSWs (Fig. 4H–J). Altogether, these findings observed in HESN female CSWs suggest that natural immunity to HIV does not lead to increase in B-cell populations presenting immature and/or innate “activated” profiles, but may involve other populations such as mature MZ-like, such as observed in EC^16^,^17^.

### Frequencies of plasmablasts, total immunoglobulins and isotypes concentrations in the blood of HIV-uninfected CSWs, HIV-infected CSWs, and HIV-uninfected non-CSWs

**Figure 5:**
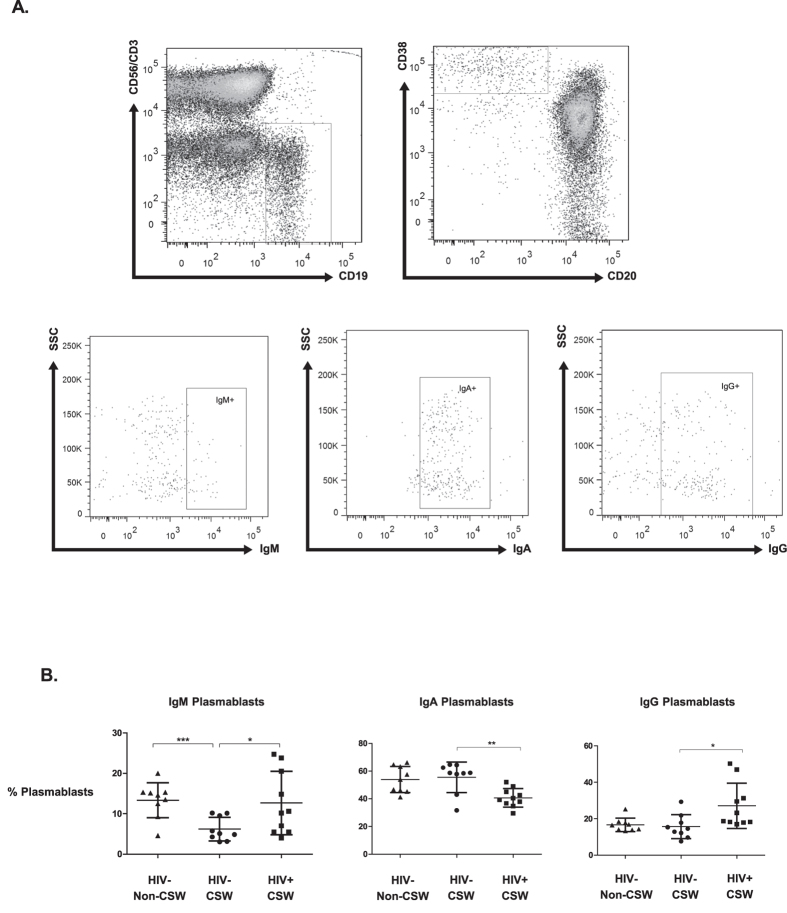
Plasmablasts frequencies in the blood of HIV-uninfected non-commercial sex workers (CSWs), HIV-uninfected CSWs and HIV-infected CSWs. Analysis by flow-cytometry was performed as follows: (**A**) Cells were gated on live PBMCs and then on total B-cells (CD19^+^CD3^−^CD56^−^). Plasmablasts were gated on CD20^-^CD38^++^ B-cells and further characterized as IgM^+^, IgA^+^ or IgG^+^. Quadrants were set based on the expression values obtained with fluorescence minus one (FMO) and isotype controls. (**B**) The frequencies (%) of IgM^+^ (left panel), IgA^+^ (middle panel) and IgG^+^ (right panel) plasmablasts are relative to total plasmablasts. The mean events gated for these populations are in the range of: CD19^+^CD20^−^CD38^++^ total plasmablasts (238 ± 112), IgM+ (25 ± 10), IgA+ (120 ± 50), IgG+ (45 ± 30). Representative FMO staining controls can be viewed in Supplementary Fig. S5. Plasmablast percentages were compared with the Mann-Whitney U tests for pair-wise comparisons between HIV-uninfected CSWs and the two other groups. Significance levels are shown as *(p < 0.05), **(p < 0.01), ***(p < 0.001).

**Figure 6:**
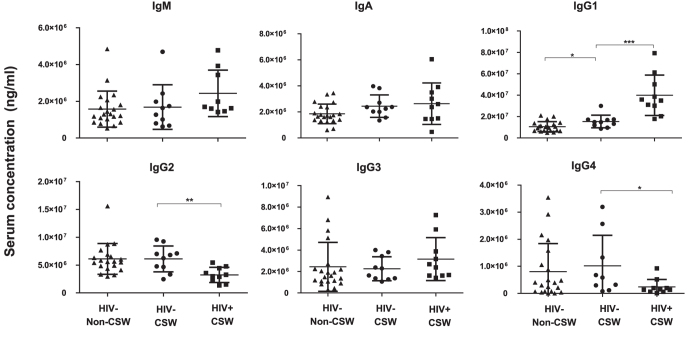
Immunoglobulin isotypes concentrations in the blood of HIV-uninfected non-commercial sex workers (CSWs), HIV-uninfected CSWs and HIV-infected CSWs. Serum immunoglobulin isotypes concentrations (ng/ml) as mean ±SEM were compared with unpaired T test for pair-wise comparisons between HIV-uninfected CSWs and the two other groups. Significance levels are shown as *(p < 0.05), **(p < 0.01), ***(p < 0.001).

HIV-uninfected CSWs had lower frequencies of blood IgM^+^ and IgG^+^ plasmablasts but higher percentages of IgA^+^ plasmablasts when compared to HIV-infected CSWs (Fig. 5A–C). The relative frequencies of IgA^+^ and IgG^+^ plasmablasts were similar between the HIV-uninfected CSW and non-CSW groups. However, HIV-uninfected CSWs had lower frequencies of IgM^+^ plasmablasts than in HIV-uninfected non-CSWs. The evaluation of total serum immunoglobulin and isotypes profile demonstrated that HIV-uninfected CSWs had lower concentration of IgG1, but higher concentration of IgG2 and IgG4 compared to HIV-infected CSWs (Fig. 6). Overall, these observations are consistent with a seemingly preserved/controlled B-cell compartment in HESN CSWs.

## Discussion

Although HIV-uninfected CSWs had the lowest levels of BLyS in their serum, they presented greater frequencies of BLyS expressing cells in their blood when compared to both HIV-infected CSWs and HIV-uninfected non-CSWs. However, HIV-infected CSWs presented the highest levels of BLyS cell surface expression. Altogether these findings suggest that natural immunity to HIV, as encountered in HIV-uninfected (HESN) CSWs, may involve relatively high frequencies of BLyS expressing cells while preserving homeostatic regulation of its cell surface expression level and soluble release. Recent studies have shown that in contrast to mDCs, plasmacytoid DCs exposed to HIV *in vitro* upregulate BLyS cell surface expression without release, and that this may depend on cellular intrinsic factors^22^. Such a regulation might not only be cell population restricted but may also be influenced by the overall inflammatory status of the host. Therefore, the low-inflammatory response we have previously described in these HIV-uninfected CSWs^13^ may be linked to the modulation of the intracellular machinery leading to BLyS expression and/or release. As to whether these are related to advantageous genetic polymorphisms remains to be established.

The relatively higher levels of BLyS observed in the serum of HIV-infected CSWs is consistent with previous reports by us and others showing that BLyS expression is increased in the context of HIV disease, and not fully restored following therapy^16^,^23^.This is likely due to direct and indirect factors associated with HIV infection. Indeed, soluble HIV-Nef can directly modulate BLyS membrane expression and soluble release by monocyte derived DCs (mo-DCs)^19^, and HIV-Env has been shown to upregulate BLyS expression by macrophages^24^. Furthermore, BLyS has been shown to be directly induced by type I IFNs^25^,^26^. Moreover, the elevated inflammatory status we have previously described in HIV-infected CSWs^3^,^13^ as well as elements of microbial translocation, such as LPS (Supplementary Fig. S1), are known to up-regulate BLyS expression and release^19^,^27^. However, blood sCD14 levels were similar between the HIV-infected and HIV-uninfected groups (Supplementary Fig. S1), suggesting that these African women might be exposed to microbial factors^28^ other than HIV that can also lead to inflammation and microbial translocation, through diarrhea for example. This may help explain why soluble levels of BLyS measured in the serum of control HIV-uninfected non-CSWs are comparable to those observed in HIV-infected CSWs, and may suggest that these individuals have inflammatory/infectious conditions other than HIV favoring soluble release of BLyS, as we did not find increased expression on the surface of blood cells we analyzed from these individuals. Albeit, our sample processing method did not allow us to assess expression by neutrophils, which are a substantial source of BLyS^29^.

In contrast to HIV-uninfected CSWs, and consistent with previous findings by us and others^15^,^30^,^31^, the high BLyS levels found in the blood of HIV-infected CSWs were concomitant with elevated serum IgG1 and major B-cell dysregulations, such as increased activation and possibly pre-exhausted profiles and a decrease in switched memory. Furthermore, in agreement with the high prevalence of IgG^+^ plasmablasts recently described in the blood of HIV-infected individuals^32^, we found that the majority of plasmablasts in HIV-infected CSWs were IgG^+^, whereas IgA^+^ plasmablasts were found predominantly in HIV-uninfected CSWs and controls, likely reflecting homeostatic requirements at mucosal interfaces such as the GALT^32^. The high frequencies of IgG^+^ plasmablasts and IgG1 hyperglobulinemia in the blood of HIV-infected CSWs is consistent with polyclonal B-cell activation found in chronic viral infections. However, we noticed a relative reduction in serum IgG2 in these individuals when compared to HIV-uninfected CSWs, which could reflect an increased trigger/need for IgG1 or perhaps IgG2 class switch interference by HIV factors such as Nef 33,34.

The fact that relative frequencies of “mature” MZ-like B-cells were reduced in the blood of both HIV-uninfected and HIV-infected CSW groups may reflect chronic exposure to HIV at peripheral mucosal entry and/or replication sites, and recruitment of this cell population and its involvement in immunity against HIV. We have previously observed the reduction of this population in the blood of HIV-infected slow progressors and EC^16^, and recent observations demonstrate that “mature” MZ-like B-cells from these individuals present greater migration index in response to CXCL12 and CCL25^35^. Whether there is a genetic relationship with the reduced frequencies of mature MZ-like B-cells we observe warrants further investigation. However, in contrast to HIV-uninfected CSWs, HIV-infected CSWs presented increased frequencies of MZ-like B-cells with an activated and precursor profile. This is likely related to the over-expression of BLyS found in HIV-infected CSWs, which favors expansion and activation of innate B-cell populations, which are likely to support over-representation of polyreactive at the expense of high affinity HIV-specific eradicative antibody (Ab) responses. Altogether, our findings in HESN female CSWs suggest that natural immunity to HIV does not lead to increase in B-cell populations presenting an immature and/or innate “activated” possibly “pre-exhausted” phenotype, but may involve other populations such as mature MZ-like and their recruitment to peripheral sites.

The B-cell phenotype we describe in the blood of HIV-uninfected CSWs likely reflects an active but regulated immune response against HIV, and is reminiscent of that we reported for EC^16^,^17^. Whereby, upregulation of BLyS seems required, but regulated as to prevent deleterious effects. The fact that we find decreased frequencies of monocytes and mDCs in the blood of both HIV-uninfected and HIV-infected CSW groups would be consistent with recruitment of these populations to peripheral sites in battling against HIV^18^,^36^. Furthermore, we find that an increased percentage of monocytes and mDC express BLyS in the blood of HIV-uninfected CSWs. In a murine HIV-transgenic model, we reported that mDCs accumulated in enlarged MZ areas^37^,^38^, likely promoting expansion of the MZ B-cell pool through cellular interactions^39^,^40^. DC surface expression may be more efficient in targeting and delivering BLyS signals to populations such as MZ-like B-cells, which express BLyS receptor TACI especially permissive to the action of BLyS in its 60 mer soluble or membrane-bound form^41^. In fact, MZ B-cells have been shown to express the short isoform of TACI, which activation leads to rapid differentiation into plasma cells^42^. We believe a regulated scheme of DC/BLyS/innate B-cell interactions could be involved in natural immunity against HIV.

As such, the recent characterization of transient Gp41-specific IgA in mucosal genital fluids from patients within the first weeks after HIV transmission, suggest these Abs might have originated from first-line B-cell populations^43^. Of note, BLyS was elevated immediately preceding the appearance of these Abs. Interestingly, repeated treatment of mice with BLyS increased the MZ compartment, and generated an increased response to Env immunization and bNAbs from these animals^44^. Understanding the dynamics of BLyS and its role in homeostasis of immune responsiveness appears pivotal to the design of vaccine strategies soliciting protective B-cell responses. Based on our observations, the capacity to contain BLyS expression levels seems concomitant with natural immunity against HIV, whereas excessive BLyS may promote immune dysregulation and disease progression^45^. However, because of the cross-sectional design and small number of samples analyzed herein, the present study cannot address whether low levels of blood BLyS and MZ-like B-cells have a protective role against HIV infection. Nevertheless the findings reported herein are in line with growing evidence suggesting that first-line B-cell responses are involved in the battle against HIV^46^, and with the importance of MZ type B-cells in health and disease^47^.

## Methods

### Subjects

Female CSWs were recruited through a dedicated sex worker clinic in Cotonou, Benin. Non-CSW HIV negative community control subjects at low risk for exposure were enrolled from a separate general health clinic in Cotonou. Women were invited to participate in the study as they attended clinics. Women under 18 years old, pregnant or menstruating were excluded from the study. Recruited women were asked to answer a questionnaire about their age, duration of prostitution (if applicable), number of clients per week (if applicable), sexual behavior, vaginal douching practices, and use of condom or hormonal contraceptive. Subjects underwent vaginal examination by a physician and vaginal specimens were used for diagnosis of candidiasis, trichomoniasis and bacterial vaginosis by microscopic examination and Herpes simplex virus (HSV) infection by polymerase chain reaction (PCR). Endocervical swabs were obtained for the detection of Neisseria gonorrhea and Chlamydia trachomatis (BD ProbeTec Et system). Blood samples were collected and tested for HIV status, diagnosis of syphilis and progesterone levels. Subjects with fever, visible vaginal inflammation and infection other than HIV were excluded from the study. HIV-1 positivity was defined by the presence of HIV-1 specific IgG tested with Vironostika HIV Uni-Form II Ag/Ab (Organon Teknika, Boxtel,The Netherlands). Non-reactive samples were considered HIV seronegative, whereas reactive samples were tested with Genie II HIV-1/HIV-2 (Bio-Rad, Hercules, CA). Genie II dually reactive samples (to HIV-1 and HIV-2) and discordant samples (Vironostika reactive/Genie II (non-reactive) were further tested by INNO-LIA HIV I/II Score (Innogenetics NV,Technologiepark 6, Gent, Belgium). For the present study we selected samples from 10 HIV-1-uninfected and 10 treatment-naïve HIV-1-infected CSWs, and 21 HIV-1-uninfected non-CSW control subjects from the general population. CD4^+^ blood T-cell counts and HIV plasma viral loads are provided for HIV-infected CSWs in Supplementary Fig. S2. None of these women were injecting drug users. The three study groups were all in the follicular phase of their menstrual cycle, not taking oral contraception, had no co-infections, bacterial vaginosis, trichomoniasis or candidiasis.

### Ethics statement

Written informed consent was obtained from all subjects who participated in the study. The investigation and methods reported in this paper were approved and carried out in accordance with guidelines and regulations by the Comité National Provisoire d’Éthique de la Recherche en Santé in Cotonou and the CHUM Research Ethics Committee.

### Sample collection and preparation

Peripheral blood mononuclear cells (PBMCs) were isolated from whole blood by centrifugation on Ficoll gradients, washed and suspended in freezing medium (90% heat inactivated fetal bovine serum (hi-FBS), 10% DMSO) and kept in liquid nitrogen until use. Plasma and serum were kept frozen at −80 °C until use.

### Determination of blood LPS, LBP, sCD14 and soluble BLyS concentration

Serum levels of lipopolysaccharide (LPS), lipopolysaccharide binding protein (LBP) and soluble CD14 (sCD14) were measured respectively with commercial Enzyme-linked immunosorbent assay (ELISA) kits by CUSABIO (Wuhan, China), Hycult Biotech (Plymouth Meeting, USA), and R&D systems (Minneapolis, USA) according to manufacturers’ protocol. Serum levels of BLyS were determined by a commercial ELISA kit by R&D systems (Minneapolis, USA).

### Determination of blood immunoglobulin and isotypes concentration

Levels of total immunoglobulin and isotypes IgG1, IgG2, IgG3, IgG4, IgM and IgA were measured in serum using the multiplex bead assay Milliplex Map Kit with human immunoglobulin isotyping Magnetic Bead panel by EMD Millipore (Billerica, USA) according to manufacturer’s protocol. Analysis was performed on a Luminex 200 System (Luminex Corporation, Austin, TX, USA).

### Evaluation of BLyS cell surface expression on T-cells, monocytes and mDC by flow cytometry

PBMCs were thawed and washed with RPMI 1640 followed by 1X PBS. A maximum of 10^6^ PBMCs per sample were used for staining. Live/Dead cell exclusion was performed using AQUA Live/Dead Fixable stain (Invitrogen Life technologies, Eugene, USA). Non-specific binding sites were blocked using a Fluorescence-activated cell sorting (FACS) buffer (1X PBS, 2% hi-FBS and 0,1% sodium azide) supplemented with 20% hi-FBS and 10ug mouse IgG (Sigma-Aldrich, St-Louis, USA) per 10^6^ cells. PBMCs were stained using the following fluorochrome conjugated mouse anti-human monoclonal antibodies: AlexaFluor 700-A anti-CD16, APC anti-CD11c, FITC anti-CD3, PE-Cy7 anti-HLA-DR, and PerCP-Cy5.5 anti-CD14 (BD-Biosciences, San Jose, CA, USA), PE anti-BLyS (ebiosciences, San Jose, CA, USA). PBMCs were fixed with 1.25% paraformaldehyde and kept at 4 °C for a minimum of 12 hours before flow cytometry analysis. A minimum of 10^5^ events per sample were acquired with a LSRFortessa (BD-Biosciences, San Jose, CA, USA) and analyzed with FlowJo7.6.3 software (TreeStar, Ashland, OR, USA). Flow-cytometry data analysis quadrants were set based on the expression values obtained with fluorescence minus one (FMO) and isotype controls. Representative FMO staining controls can be viewed in Supplementary Fig. S3.

### Evaluation of blood B-cell populations by flow cytometry

PBMCs processing, staining and analysis were performed as mentioned above. PBMCs were stained using the following fluorochrome conjugated mouse anti-human monoclonal antibodies: APC-Cy7 anti-CD10, Pacific Blue anti-CD19 (BioLegend, San Diego, CA, USA), Alexa Fluor700 anti-CD27, FITC anti-IgM, PE anti-CD21, BV650-anti-CD19, APC-H7-anti-IgG, BV605-anti-IgM (BD-Biosciences, San Jose, CA, USA), APC-anti-CD3, APC-anti-CD56, PE-Cy7-anti-CD20, PE-anti-CD38, PerCP efluor710 anti-CD1c (ebiosciences, San Jose, CA, USA), and FITC-anti-IgA (EMD Millipore, Temecula, CA, USA). Flow-cytometry data analysis quadrants were set based on the expression values obtained with fluorescence minus one (FMO) and isotype controls. Representative FMO staining controls can be viewed in Supplementary Fig. S4 and S5.

### Statistical analyses

Data from HIV-uninfected CSWs were compared separately from those of HIV-infected CSWs and HIV-uninfected non-CSWs. The statistical significance of difference between groups was determined by Fisher’s exact test for categorical variables and unpaired Student’s T-test or one-way ANOVA analysis for variance when continuous variables were normally distributed or by Mann-Whitney U test otherwise. The D’Agostino-Pearson normality test was used to determine whether the values were sampled from a Gaussian distribution. Analyses were performed using GraphPad Prism 5.00 for Windows (GraphPad Software, San Diego, California, USA).

## Additional Information

**How to cite this article**: Sabourin-Poirier, C. *et al.* Blood B Lymphocyte Stimulator (BLyS)/BAFF levels may reflect natural immunity to HIV in highly exposed uninfected Beninese Commercial Sex Workers. *Sci. Rep.* **6**, 32318; doi: 10.1038/srep32318 (2016).

## Supplementary Material

Supplementary Information

## Figures and Tables

**Table 1:** Distribution of demographic and sexual behavior characteristics in HIV-uninfected non-CSW control subjects, HIV-uninfected and HIV-infected CSWs.

	HIV-uninfected non-CSWs	HIV-uninfected CSWs	HIV-infected CSWs	p-value^a^
	N = 21	N = 10	N = 10	
Age, mean (SD), years	37.6 (9.5)	41.4 (8.3)	43.1 (10.1)	NS
Duration of sex work. mean (SD), years	NA	5.6 (1.4)	5.4 (3.3)	NS
Number of client past week, mean (SD)	NA	17.6 (14.9)	19.6 (14.3)	NS
Condom always used with clients past week	NA	8/10 (80%)	3/10 (30%)	NS
Vaginal douching	21/21 (100%)	10/10 (100%)	10/10 (100%)	NS

CSW, commercial sex workers; HIV, human immunodeficiency virus; N, number of participants; NA, non-applicable; NS, non-significant; SD, standard deviation.

^a^p-value for the comparison across all groups were calculated with one-way ANOVA analysis for variance of the age; Mann-Whitney U test for the duration of sex work and number of clients; Fisher’s exact test for condom use and vaginal douching.
