# Supplementary material for: Blood B Lymphocyte Stimulator (BLyS)/BAFF levels may reflect natural immunity to HIV in highly exposed uninfected Beninese Commercial Sex Workers
Source: Sci Rep. 2016 Aug 26;6:32318. doi: 10.1038/srep32318 (PMC4999816; doi:10.1038/srep32318)
Supplement: Supplementary Information [file srep32318-s1.pdf]

Blood B Lymphocyte Stimulator (BLyS)/BAFF levels may reflect natural immunity to HIV in highly exposed uninfected Beninese Commercial Sex Workers.

Catherine Sabourin-Poirier[1,2], Lyvia Fourcade[1,2], Annie-Claude Labbé[2], Michel Alary[3,4], Fernand Guédou[5], Johanne Poudrier[1,2]*, and Michel Roger[1,2]*

Supplementary Figure S1

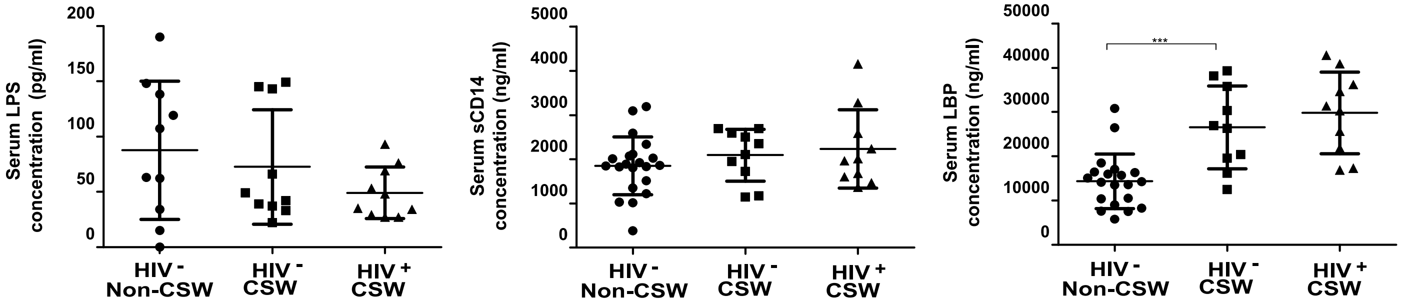

Supplementary Figure S1. Concentration of lipopolysaccharide (LPS), lipopolysaccharide binding protein (LBP) and soluble CD14 (CD14s) in the blood of HIV-1 uninfected non-commercial sex workers (CSWs), HIV-1 uninfected CSWs and HIV-1 infected CSWs Serum concentrations as mean ± SEM (pg/ml), (ng/ml) and (ng/ml) respectively, were compared with unpaired T test. Significance levels are shown as ***($p<0.001$).

Supplementary Figure S2

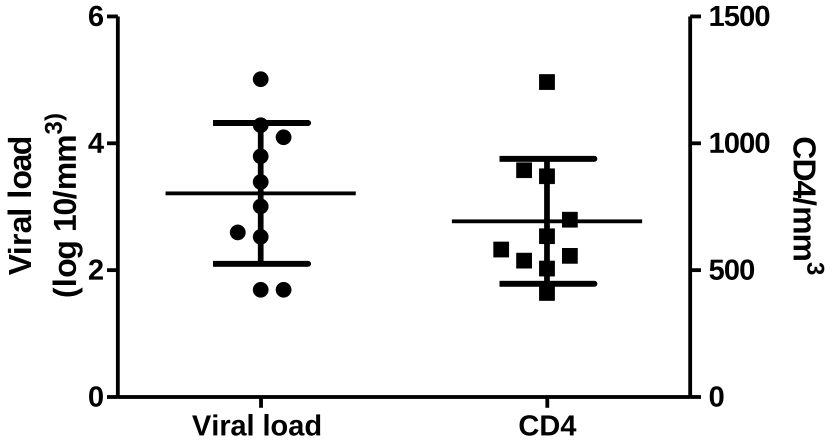

Supplementary Figure 2. Plasma viral loads and blood CD4+ T-cell counts of HIV-infected commercial sex workers (CSWs). CD4+ T cell counts (cell/mm^3) and viral loads (log^{10} copies/ml) as mean ±SEM.

Supplementary Figure S3

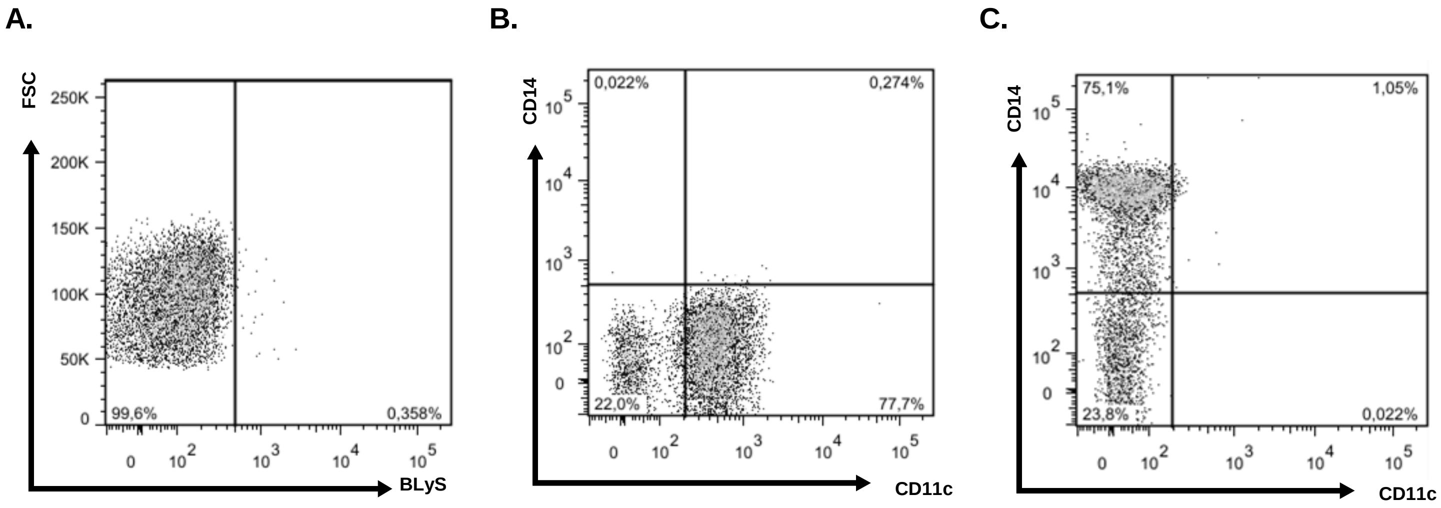

Supplementary Figure S3. Representative fluorescence minus one (FMO) staining controls for Figures 2 and 3. FMO of BLyS (A), FMO of CD14 vs CD11c positive staining (B), and FMO of CD11c vs CD14 positive staining (C) on gated live HLA-DR+CD3- APCs.

Supplementary Figure S4

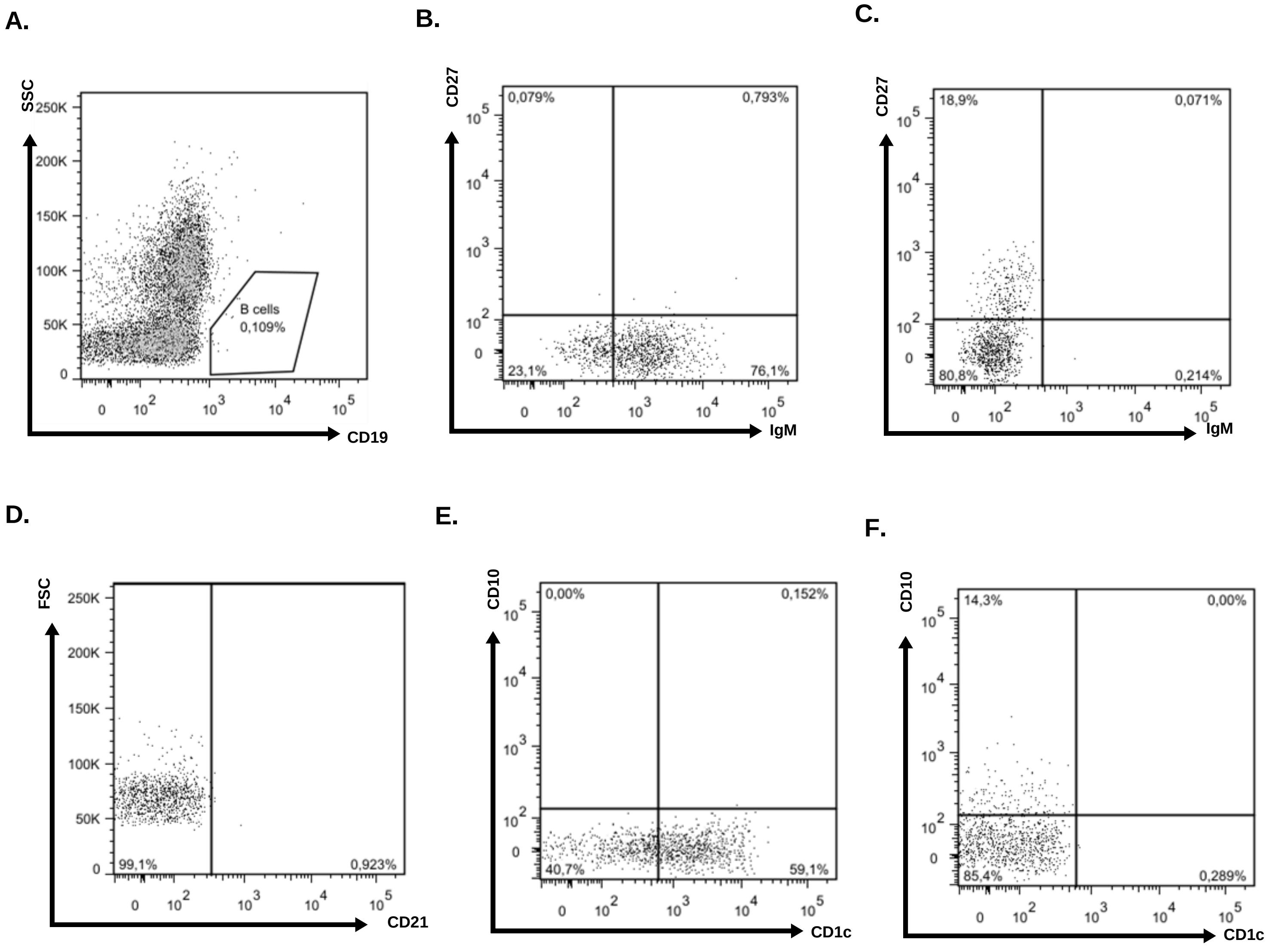

Supplementary Figure S4. Representative fluorescence minus one (FMO) staining controls for Figure 4. FMO of CD19 on live gate (A), FMO of CD27 vs IgM positive staining (B), FMO of IgM vs CD27 positive staining (C), and FMO of CD21 (D) on gated live CD19+ B-cells. FMO of CD10 vs CD1c positive staining (E),and FMO of CD1c vs CD10 positive staining (F) on gated live CD27+IgM+ B-cells.

Supplementary Figure S5

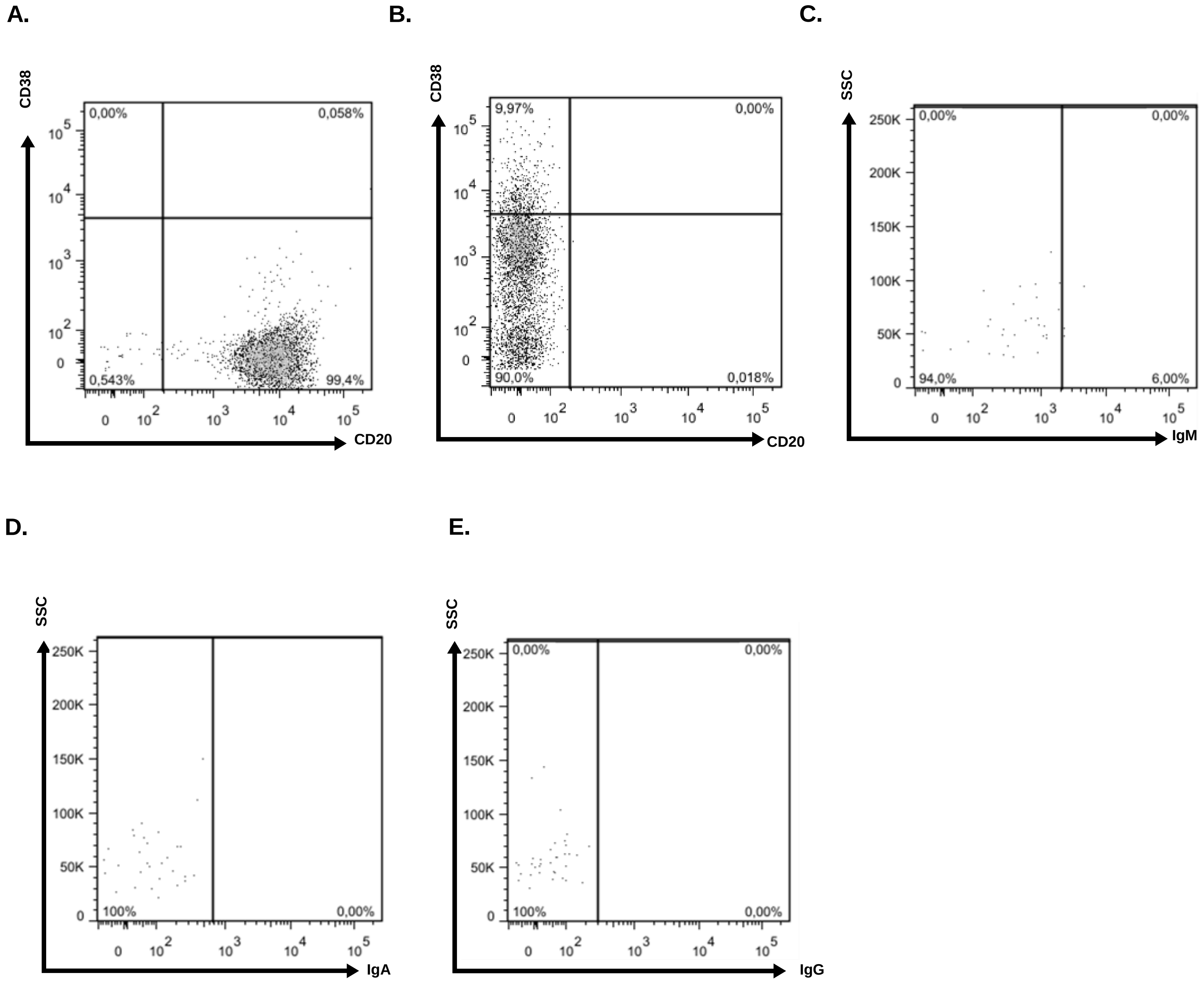

Supplementary Figure S5. Representative fluorescence minus one (FMO) staining controls for Figure 5. FMO of CD38 vs CD20 positive staining (A), and FMO of CD20 vs CD38 positive staining (B) on live gated B-cells. FMO of IgM (C), FMO of IgA (D), FMO of IgG on gated live CD38++CD20- plasmablasts.
